# Sulfamethoxazole-Altered Transcriptomein Green Alga *Raphidocelis subcapitata* Suggests Inhibition of Translation and DNA Damage Repair

**DOI:** 10.3389/fmicb.2021.541451

**Published:** 2021-07-19

**Authors:** Jiahua Guo, Yibo Zhang, Jiezhang Mo, Haotian Sun, Qi Li

**Affiliations:** ^1^Shaanxi Key Laboratory of Earth Surface System and Environmental Carrying Capacity, College of Urban and Environmental Sciences, Northwest University, Xi’an, China; ^2^School of Environmental Science and Engineering, Huazhong University of Science and Technology, Wuhan, China; ^3^Department of Chemistry, City University of Hong Kong, Hong Kong, China

**Keywords:** sulfonamide antibiotic, RNA-seq, DNA replication and repair, chlorophyll *a* synthesis, molecular mechanism

## Abstract

Occurrence of sulfonamide antibiotics has been reported in surface waters with the exposures ranging from < 1 ng L^–1^ to approximately 11 μg L^–1^, which may exert adverse effects on non-target algal species, inhibiting algal growth and further hindering the delivery of several ecosystem services. Yet the molecular mechanisms of sulfonamide in algae remain undetermined. The aims of the present work are: (1) to test the hypothesis whether sulfamethoxazole (SMX) inhibits the folate biosynthesis in a model green alga *Raphidocelis subcapitata*; and (2) to explore the effects of SMX at an environmentally relevant concentration on algal health. Here, transcriptomic analysis was applied to investigate the changes at the molecular levels in *R. subcapitata* treated with SMX at the concentrations of 5 and 300 μg L^–1^. After 7-day exposure, the algal density in the 5 μg L^–1^ group was not different from that in the controls, whereas a marked reduction of 63% in the high SMX group was identified. Using the adj *p* < 0.05 and absolute log_2_ fold change > 1 as a cutoff, we identified 1 (0 up- and 1 downregulated) and 1,103 (696 up- and 407 downregulated) differentially expressed genes (DEGs) in the 5 and 300 μg L^–1^ treatment groups, respectively. This result suggested that SMX at an environmentally relevant exposure may not damage algal health. In the 300 μg L^–1^ group, DEGs were primarily enriched in the DNA replication and repair, photosynthesis, and translation pathways. Particularly, the downregulation of base and nucleotide excision repair pathways suggested that SMX may be genotoxic and cause DNA damage in alga. However, the folate biosynthesis pathway was not enriched, suggesting that SMX does not necessarily inhibit the algal growth via its mode of action in bacteria. Taken together, this study revealed the molecular mechanism of action of SMX in algal growth inhibition.

## Introduction

Sulfonamides are synthetic drugs used in antibacterial, antidiabetic, diuretic, anticonvulsant, and herbicidal applications. Owing to the widespread use and improper handling of antibiotics, concerns have been raised over the potentially ecological risks of sulfonamides present in the environment (Michael et al., 2013; Bai and Acharya, 2016; Vila-Costa et al., 2017). Sulfamethoxazole (SMX) is a commonly used sulfa antibiotic designed to treat respiratory, urinary, skin, and gastrointestinal infections, as well as in aquaculture and livestock farming to promote animal health (Chen and Xie, 2018). In China, the annual total usage of SMX in 2013 was approximately 313 tons (Zhang et al., 2015). Since SMX cannot be entirely absorbed and/or metabolized in the human body, a considerable proportion of SMX is excreted into the sewer system as feces and urine (Sarmah et al., 2006; Tappe et al., 2013). However, the traditional activated sludge process in the sewage treatment plants is not designed for the efficient removal of emerging pollutants such as sulfonamides. Consequently, the unchanged SMX included in the effluent is released into the freshwater ecosystem. Furthermore, it is noteworthy that SMX is considered as pseudo-persistent in the environment because of its constant consumption and release (Kümmerer, 2009). In a study analyzing the pharmaceuticals in more than 100 river samples from 27 European countries, SMX has been detected in concentrations ranging from < 1 ng L^–1^ to approximately 4.1 μg L^–1^ (Loos et al., 2009). Thus, the occurrence of SMX in the wild is probable to adversely affect the health of aquatic species.

Algae, widely distributed in the aquatic ecosystems, are the primary producers of ecosystems, supplying food for higher trophic level animals (Lai et al., 2009). Given that algae deliver several provisioning and supporting services such as biomass outputs and oxygen production during photosynthesis (ca. 50% of all oxygen production), pollutants-induced damage in algae may pose a threat to the stability of the aquatic ecosystem (Chapman, 2010; Maltby et al., 2018). Therefore, algal toxicity testing is a prerequisite for hazard assessment during the marketing authorization process of chemicals with antimicrobial properties (EMA, 2018). Green alga *R. subcapitata*, formerly known as *Pseudokirchneriella subcapitata* or *Selenastrum capricornutum*, is recommended as a standard test organism in view of its high sensitivity and rapid growth rate, which enables the evaluation of chemical effects on several generations. The traditional algal toxicity testing emphasized only on the acute toxicological effects such as growth inhibition, relying on the unrealistic exposure levels that may overlook the sublethal effects. For example, exposure to SMX was reported to inhibit the growth of several algal species, such as *R. subcapitata*, *Synechococcus leopolensis*, *Scenedesmus vacuolatus*, and *Scenedesmus obliquus* with the EC50 values of 0.52, 0.027, 1.54, and 0.11 mg L^–1^, respectively (Ferrari et al., 2004; Isidori et al., 2005; Białk-Bielińska et al., 2011; Xiong et al., 2019). For *Scenedesmus obliquus*, SMX at the concentration of 0.25 mg L^–1^ or higher reduced the dry cell weight, content of photosynthetic pigments, fatty acid methyl ester composition, and so forth. The ecotoxicological effects of sulfonamides on microalgae have been reported previously (Białk-Bielińska et al., 2011; Borecka et al., 2016). However, to our knowledge, a comprehensive evaluation of the tolerance mechanisms of microalgae during the toxicity test of such organic contaminants is not well established (Xiong et al., 2019). Recent ecotoxicological studies have shifted from the apical endpoints to molecular levels chronically altered by chemicals at environmentally relevant concentrations, as changes at the molecular level may have a long-term ecological implication (Vannini et al., 2011). In particular, transcriptomic analysis that quantitatively analyzes the genome-wide gene expression can identify biological pathways disturbed by the presence of toxic substances. However, this approach has not been applied to evaluate the effects of SMX on algal species.

The SMX is a bacteriostatic antibiotic that acts as a structural analog of the substrate *p*-aminobenzoic acid (pABA), inhibits dihydropterin synthase, and blocks the synthesis of folic acid in bacteria (Brain et al., 2008). In light of a high degree of homology conserved in chloroplast and bacteria, such mode of action may occur and exert adverse effects SMX on aquatic plants (Basset et al., 2004; Manuell et al., 2004; Grenni et al., 2019). It is reported that SMX could significantly inhibit the photosynthetic progress including primary photochemistry, electron transport, photophosphorylation, and carbon assimilation (Liu et al., 2011b). In the present study, an evaluation on the gene expression profiles in *R. subcapitata* treated with SMX was performed. Here, an environmentally relevant concentration of 5 μg L^–1^ and an exposure of 300 μg L^–1^ known to cause approximately 50% growth inhibition in the preliminary test were chosen for toxicity testing. It is hypothesized that SMX treatment may lead to the differentially expressed genes (DEGs) enriched in the biological pathways of folate and chlorophyll biosynthesis. The objectives of the present study were: (1) to evaluate the effects of SMX at an environmentally relevant concentration on the transcriptomic profile in *R. subcapitata*; and (2) to uncover the toxicological mechanism underlying the inhibited growth induced by SMX.

## Materials and Methods

### Chemicals

Sulfamethoxazole (≥98.0% purity, HPLC grade; CAS no.723-46-6) was purchased from Yuanye Bio-Technology Co., Ltd. (Shanghai, China). Methanol (CAS no. 75-56-1; HPLC ≥ 99.9%) and acetonitrile (CAS no. 75-25-8; HPLC ≥ 99.9%) were purchased from Tedia (United States). Formic acid (HPLC grade) was obtained from Komeo Chemical Reagent Co., Ltd. (Tianjin, China). Atrazine D5 (CAS no. 163165-75-1) was obtained from J&K Scientific (Beijing, China). Sulfamethoxazole-13C6 (≥99.4% purity; CAS no. 1196157-90-0). Ultrapure water with a resistivity of at least 18.2 MΩ was prepared using a Milli-Q purification system (Millipore, United States). Other chemicals used for cultural medium preparation were at least reagent grade.

### Algal Cultures

The *R. subcapitata* (FACHB-271) was obtained from the Freshwater Algae Culture Collection at the Institute of Hydrobiology, Chinese Academy of Sciences (Wuhan, China). The alga was cultured in the laboratory following the OECD guideline No. 201 (OECD, 2011). The alga was individually inoculated in 250 mL Erlenmeyer flasks containing 100 mL Blue-Green (BG11) medium (pH 7.1) with the initial cell density of 1 × 10^4^ cells mL^–1^. To avoid contamination, the Erlenmeyer flasks capped with air-permeable stoppers made of cotton and muslin were autoclaved at 121°C for 30 min prior to its usage. The alga was cultured at 22 ± 2°C under 75 μmol m^–2^ s^–1^ in an illumination chamber (DongNan Instrument Co., Ltd., China). During the cultivation, all flasks were shaken three times a day to prevent cells from adhering to the walls of the flask. The cell density was estimated using a hemocytometer (Hausser Scientific, United States) under the microscope (Phenix Optical Technology Co., Ltd., China) and a growth curve was plotted to determine the exponential phase (usually over 2–4 days of cultivation). Algae at this stage were employed for further testing.

### Procedures for the Growth Inhibition Test

Growth inhibition test was undertaken in accordance with the OECD 201 guideline. Prior to use, all glassware used in the tests were autoclaved at 121°C for 30 min. The antibiotic solutions at the concentrations of 0 (control), 0.005 (low), and 0.3 (high) mg L^–1^ were prepared with the culture medium and filtered with a 0.22 μm sterilized syringe-driven filter. The alga at the exponential phase was inoculated to each treatment group, in triplicate, at the density of 1 × 10^4^ cells mL^–1^. All the operations were performed on a clean bench (Biobase Biotech Co., Ltd., China).

Algae were placed in the illumination incubator with the same conditions as what was used for the algal culture. The test lasted for 7 days, and a 0.5 mL of each algal suspension was withdrawn at regular intervals (days 0, 2, 4, and 7) to estimate the cell density for plotting the growth curve. At day 7, subsequent to the centrifugation of algal suspensions at 7,100 g for 15 min, the harvested biomass was snap frozen in liquid nitrogen and stored at −80°C prior to RNA extraction.

### RNA-Seq

Total RNA in the alga was extracted using the Trizol method. The concentration and purity were determined using the NanoDrop 2000 (Thermo Scientific, Waltham, MA, United States). The integrity (RIN value) of total RNA measured by the 2100 Bioanalyzer (Agilent, United States) were all above 8. Next-generation sequencing was conducted for all the total RNA samples using the Illumina NovaSeq (Shanghai Personal Biotechnology Co., Ltd., China). Paired-end method was applied for RNA sequencing and the read length was 150 base pair. The mapped reads for all the samples ranged from 50,278,609 to 65,134,610, and the transcriptome coverage varied from 94.51 to 95.73%.

### RNA-Seq Data Analyses

The raw reads in FASTQC (v. 0.11.9) format were initially generated (Andrews, 2010), followed by pruning the 3′ end_band adapter sequence, and removing the low-quality values (QV < 20). Regions in the sequencing files were pruned using the Cutadapt (v 1.1). The trimmed reads were mapped to the reference genome *Raphidocelis subcapitata* 1.0 (GCA_003203535.1) using the Hisat2 (v. 2.1.0). We used HTSeq (v. 0.11.1) in R (v. 3.6.3) to estimate the read count for each gene (Anders et al., 2014), which was further normalized to fragments per kilobase of transcript per million mapped reads (FPKM) used as the gene expression level. To examine the consistency of three replicates in each group, Pearson correlation coefficients representing the correlation of gene expression levels between samples were estimated. Samples were clustered by principal component analysis (PCA) in the “DESeq” package (v1.39.0) in R (v. 3.6.3) (Anders and Huber, 2010).

To identify the genes that were differentially expressed in the SMX-treated group relative to the controls, the “DESeq” package in R was used to screen the DEGs, with a cutoff of | log_2_ fold change (FC)| > 1 and adj *p*-value < 0.05 (Root et al., 2020). A volcano plot visualizing the expression of DEGs was created using the “ggplots 2” package (v. 3.3.2). A heatmap was further produced using the centered and scaled FPKM values of DEGs in the “Pheatmap” package (v.1.0.12) (Wu et al., 2020). The DEGs in the SMX-treated group was submitted for the functional enrichment analyses of gene ontology (GO) and kyoto encyclopedia of genes and genomes (KEGG) pathway. The GO enrichment analysis was performed in the “TopGO” package (v.2.40.0) to annotate DEGs in terms of molecular function (MF), biological process (BP), and cell component (CC) (Alexa and Rahnenfuhrer, 2020). A *p*< 0.05 was considered as a cutoff for identifying the significantly enriched GO terms and biological pathways. The KEGG pathway analyses were performed in “clusterprofiler” (v.3.16.1), DEGs with a *p* < 0.05 was used as a cutoff to determine the enriched pathways. The raw sequencing data were deposited into the National Omics Data Encyclopedia (NODE) under accession number OEP001463^1^.

### Quantitative Real-Time PCR

To validate the gene expression profile obtained from RNA-seq, the expression levels of magnesium chelatase subunit D (*chlD*) and magnesium-protoporphyrin O-methyltransferase (*chlM*) involved in the porphyrin and chlorophyll metabolism pathway, and DNA replication licensing factor MCM2 (*mcm2*) and proliferating cell nuclear antigen (*pcna*) participated in the DNA replication process were measured by quantitative real-time polymerase chain reaction (qRT-PCR). The expression levels of these genes were normalized to a housekeeping gene ubiquitin-conjugating enzyme E2 G1 (*ubc*). Details on the PCR process can be found in Supplementary Material.

### Antibiotic Analyses

The initial exposure levels of SMX used for the toxicity testing were confirmed by liquid chromatography tandem-mass spectrometry (LC-MS/MS, Agilent, United States) analysis coupled with C18 column (ZORBAX eclspse plus column 600 bar, 3 mm × 100 mm × 1.8 μm). Details on the solid phase extraction procedures, instrumental setting, and method validation can be found in Supplementary Tables 1–3. As the variation between the measured and nominal concentrations was less than 20%, the nominal concentration of each group was applied for further investigation.

### Statistical Analyses

At day 7, differences in the growth between the SMX-treated and control groups were statistically evaluated by one-way analysis of variance (ANOVA) followed by Dunnett’s *post hoc* tests (GraphPad Prism 8). The correlation between transcriptomics and qRT-PCR assays was determined for *chlD*, *chlM*, *mcm2*, and *pcna* using Pearson correlation analysis. Here, a *p* < 0.05 was taken as having a significant difference.

## Results

### Effects of SMX on Algal Growth

**FIGURE 1:**
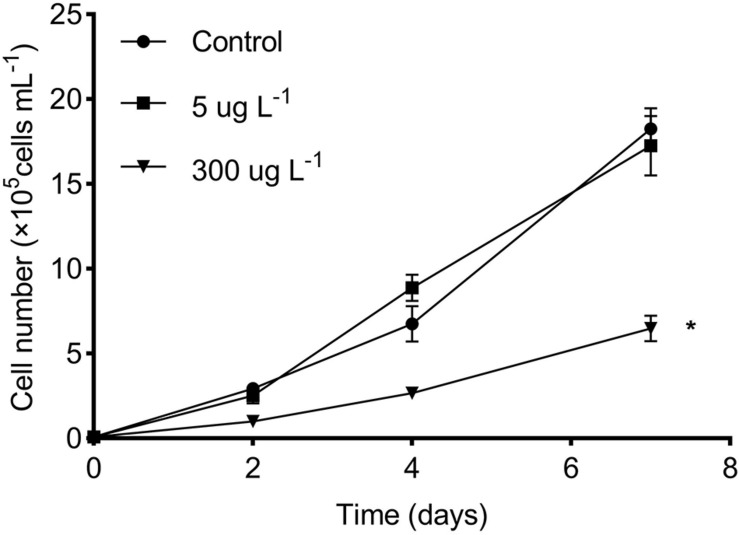
Effects of SMX concentration on microalgal growth of *R. subcapitata*. The asterisk (*) indicates significant difference (*p* < 0.05) between the control and SMX treatments. Data are presented as mean values ± standard deviation (*n* = 3).

The growth curves of *R. subcapitata* in the control and SMX-treated groups are presented in Figure 1. The algal density in the low group was not different from that in the controls, whereas there was a marked reduction in the high SMX group. In particular, a growth inhibition rate of approximately 63% was attained in the high treatment group at day 7.

### Differentially Expressed Genes

**FIGURE 2:**
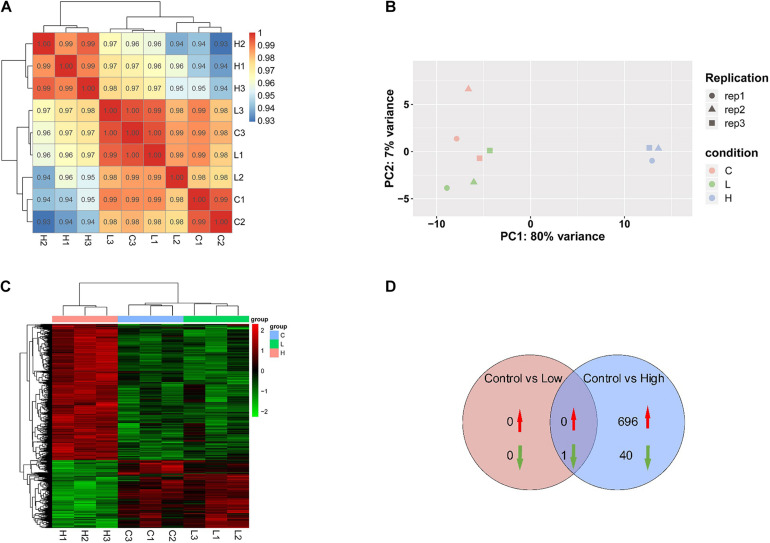
Transcriptomic profile of *R. subcapitata* treated with SMX for 7 days. **(A)** Correlation analysis of gene expression patterns control and SMX-treated groups. **(B)** Principal component analysis (PCA) of FPKM profiles of DEG. **(C)** A heatmap of centered and scaled FPKM values of DEGs in the controls and SMX-treated group. **(D)** Venn diagram of DEGs in each group.

According to transcriptomic analysis, 13,383 transcripts were identified in *R. subcapitata*. A high correlation among the replicates in each treatment group was detected, with the correlation coefficients no less than 0.98 (Figure 2A). The PCA analysis indicated that the gene expression pattern in the controls resembled that in the low concentration, suggesting that no markedly detrimental effects were caused by SMX at the environmentally relevant concentration (Figure 2B). Nonetheless, samples in the high group were clearly distinguishable from those in the control and low treatment groups, suggesting that SMX at the high concentration may lead to adverse outcomes, which agrees with the remarkably inhibited growth of *R. subcapitata*.

**FIGURE 3:**
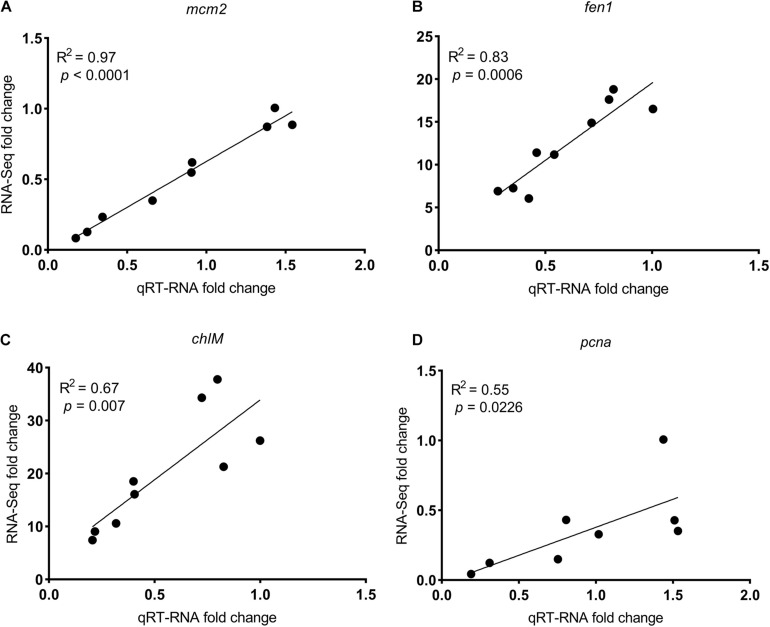
The correlation between fold changes (SMX-treated groups relative to control) in mRNA levels determined by qRT-PCR and RNA-seq of genes **(A)** *mcm2*, **(B)** *fen1*, **(C)** *chlM*, and **(D)** *pcna*. *mcm2*, DNA replication licensing factor MCM2; *fen1*, flap endonuclease-1; *pcna*, proliferating cell nuclear antigen; *chlM*, magnesium-protoporphyrin O-methyltransferase.

Exposure to SMX at the low and high concentrations gave rise to 1 (0 up- and 1 downregulated) and 1,103 (696 up- and 407 downregulated) genes that was differentially expressed in *R. subcapitata* (Figures 2C,D and Supplementary Figure 1). To ascertain the NGS results, expression levels of four DEGs, *mcm2*, *fen1*, *pcna*, and *ch1M*, were validated by qRT-PCR (Figure 3). The FPKM values were highly correlated with the mRNA expression levels measured by qRT-PCR, with *R*^2^ = 0.97, *p* < 0.0001; *R*^2^ = 0.83, *p* < 0.0006; *R*^2^ = 0.55, *p* = 0.0226; and *R*^2^ = 0.67, *p* < 0.007 for *mcm2*, *fen1*, *pcna*, and *ch1M*, respectively. The mRNA expression levels measured by qRT-PCR were concordant with the NGS data, indicating that the mRNA expression profiles in *R. subcapitata* provided by NGS were quantified with confidence.

### Gene Ontology and Functional Pathway

The GO analysis was performed to explore the roles of DEGs in the CC, MF, and BP, where the top 10 enriched GO terms in the low and high SMX treatment groups were illustrated in Supplementary Figure 2. In the high treatment group, several GO terms are mainly related to DNA replication (GO:0042555 MCM complex, GO:0006260 DNA replication etc.), chlorophyll synthesis (GO:0033013 tetrapyrrole metabolic process, GO:0033014 tetrapyrrole biosynthetic process etc.), and translation processes (GO:0005730 nucleolus, GO:0015631 tubulin binding).

The present study tested the hypothesis that SMX treatment may interfere with the signaling pathways in relation to folate biosynthesis and photosynthesis. Consistent with the hypothesis, SMX at the high exposure level downregulated the genes involved in the photosynthesis-related pathways; however, enrichment of the folate biosynthesis pathway was not detected. Unexpectedly, genes involved in translation and DNA replication and repair processes were downregulated (Table 1). In the present study, the downregulated DEGs were involved in various phases of DNA replication and repair. DNA replication licensing factors (*mcm2-7*) function at the replication fork formation stage. Besides, the elongation genes consisting of the proliferating cell nuclear antigen (*pcna*), replication factor C subunit 3 (*rfc*3/5), and replication A1, 2 (*rfa1*, *rfa2*) were also decreased. Furthermore, the DNA polymerase alpha and delta catalytic subunits (*pola1*, *pold1*), ribonuclease H2 subunit C (*rnaseh2C*), and flap endonuclease-1 (*fen1*) involved in the termination stage of DNA replication were also reduced. Genes involved in photosynthesis, including chlorophyll synthesis and photosynthesis- antenna proteins, were also downregulated. Genes of synthesis of chlorophyll including Glu-tRNA reductase (*hemA*), porphobilinogen synthase (*HemB*), hydroxymethylbilane synthase (*HemC*), uroporphyrinogen decarboxylase (*HemE*), coproporphyrinogen III oxidase (*HemF*), magnesium chelatase subunitH (*chlH*), magnesium-protoporphyrin O-methyltransferase (*chlM*), vitamin chlorophyllide an 8-vinyl-reductase (*dvr*), and protochlorophyllide reductase (*por*) were downregulated. Genes involved in translation were downregulated, including complete fibrillarin (*nop1*), nucleolar protein 56 (*nop56*), GTP-binding nuclear protein Ran (*ran*), glutamyl-tRNA synthetase (*gltx*), leucyl-tRNA synthetase (*leus*), lysyl-tRNA synthetase (*lysk*), and tyrosyl-tRNA synthetase (*tyrs*), in the high SMX treatment group.

**TABLE 1:** List of key enriched pathways (*p* < 0.05) in *R. subcapitata* treated with 300 μg L^–1^ of sulfamethoxazole.

Pathway	Category	Up_gene	Down_gene	*p*-value	FDR
**Control vs. High**					
DNA replication	Replication and repair		*fen1*, *mcm2*, *mcm3*, *mcm4*, *mcm5*, *mcm6*, *mcm7*, *pcna*, *pola1*, *pold1*, *pole*, *pole2*, *pri1*, *rfa1*, *rfa2*, *rfc3/5*, *rnaseh2c*.	8.2 × 10^–13^	5.32 × 10^–11^
Mismatch repair	Replication and repair		*msh2*, *pcna*, *pold1*, *rfa1*, *rfa2*, *rfc3/5*.	0.0023	0.038
Base excision repair	Replication and repair		*fen1*, *pcna*, *pold1*, *pole*, *pole2.*	0.0067	0.088
Nucleotide excision repair	Replication and repair		*pcna*, *pold1*, *pole*, *pole2*, *rfa1*, *rfa2*, *rfc3/5*.	0.013	0.14
Porphyrin and chlorophyll metabolism	Metabolism of cofactors and vitamins		*chlD*, *chlM*, *dvr*, *gltX*, *hemA*, *hemB*, *hemC*, *hemE*, *hemF*, *hemL*, *por.*	3.45 × 10^–8^	1.1 × 10^–4^
Photosynthesis - antenna proteins	Energy metabolism		*lhca2*, *lhcb1*, *lhcb2*, *lhcb4*, *lhcb5.*	0.0018	0.038
Aminoacyl-tRNA biosynthesis	Translation	*sepsecs*	*gltx*, *lleus*, *lyss*, *tyrs*.	0.037	0.28
Ribosome biogenesis in eukaryotes	Translation	*pop1*	*gnl3*, *nop1*, *nop56*, *ran*, *Rcl1*, *utp4*.	0.047	0.31
Nitrogen metabolism	Metabolism	*cpy55*	*nirA*, *nrt*.	0.013	0.14
Glycosylphosphatidylinositol (GPI)-anchor biosynthesis	Metabolism	*pigc*, *pigl*, *pigp*		0.039	0.28

## Discussion

**FIGURE 4:**
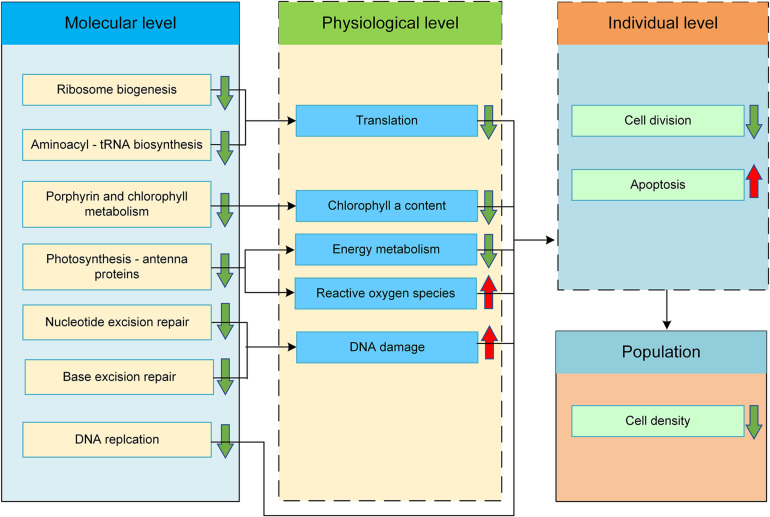
Proposed molecular mechanism of action of SMX in green alga *R. subcapitata*. Changes in the physiological and individual levels were predicted based on the enriched pathways.

A schematic diagram illustrating the molecular mechanism underlying the inhibited growth of *R. subcapitata* in response to SMX exposure was proposed (Figure 4). In the following sections, we discuss the effects of SMX on these enriched signaling pathways.

### Genes Related to DNA Replication and Repair

DNA replication is one of the most important mechanisms for cell division. Any error in the replication process, even in one base, or any disturbance in the cell cycle may cause genome instability or cell death (Fournet and Roussakis, 2018). The DNA replication process was inhibited in algae treated with external stressors such as heavy metals and oxide nanoparticles (Holzinger et al., 2014; Schiavo et al., 2016). In the high SMX treatment group, expression of a multitude of DEGs involved in the DNA replication process was repressed, suggesting that this pathway is a major target of SMX in *R. subcapitata* (Table 1). The downregulated DEGs participated in the various stages of DNA replication. The DNA replication licensing factor (*mcm2-7*), clamp proliferating cell nuclear antigen (*pcna*), replication factor C subunit 3/5 (*rfc3/5*), and replication factor (*rfa1 and 2*) are involved in replication fork formation and primary binding were decreased. The minichromosome maintenance protein complex (MCM) is a checkpoint at the stage of DNA synthesis, its expression is inhibited causing DNA damage and genome instability (Guo et al., 2020). Loading of *pcna* onto the chromatin is an important step for the initiation of DNA synthesis (Kang et al., 2019). Previous studies have shown that the inhibited expression of *pcna* gave rise to the morphology and structural changes in the cell nucleus (Li et al., 2015). During DNA replication, *pcna* needs to be opened and closed to encircle the DNA. Furthermore, *rfc* is the main rate-limiting step during *pcna* loading (Sakato et al., 2012). Therefore, the SMX-induced reduction in the *rfc* and *pcna* expression may spark the inhibition of elongation phase. DNA replication is a key event of cell proliferation, which may be permanently and/or temporarily hindered by the inhibition of fork formation and primary binding as replisome components become irretrievably inactivated (Syed et al., 2016). Defects in the initiation of DNA replication are also probable to cause apoptosis (Weinberger et al., 2005). DNA polymerase alpha subunit A (*pola1*), DNA primase small subunit (*pri1*), DNA polymerase delta subunit 1 (*pold1*), and DNA polymerase epsilon subunits 1 and 2 (*pole* and *pole2*) which played roles in the elongation process were reduced. *pola1* encodes the catalytic subunit of DNA polymerase α, which is a prerequisite for the initiation of DNA replication. Previous studies have shown that CD437 exerts its cytotoxicity by inhibiting *pola1*. *pold1* has both polymerase and exonuclease activities. Total *pold1* deficiency can lead to a high rate of DNA replication errors and result in embryonic damage that are lethal in mice (Cui et al., 2019). Ribonuclease H2 subunit C (*rnaseh2c*) and flap endonuclease-1 (*fen1*) which are indispensable during the termination phase were also reduced. The *fen1*, via its interaction with *pcna*, functions in both DNA replication and repair. The nuclease activity of *fen1* can be stimulated by *pcna* to enhance stability at the branch point and increase its chance of cutting (Craggs et al., 2013; Boehm et al., 2016). Prior to each cell division, guarantee on enough energy and materials (e.g., primer, enzymes, and deoxynucleotide) obtaining for sufficient cell growth, proper DNA replication, and cell cycle checking are necessary. Lack of enough energy and materials (Vitova et al., 2015) and DNA damage without being properly repaired (Roos and Kaina, 2013) may inhibit DNA replication. As DNA replication is a critical part of cell proliferation, the DNA replication pathway downregulated by SMX in algal cells was inferred to lead to the inhibition on the next cell division process or apoptosis, resulting in a decreased algal density.

Genomic DNA damaged by both endogenous and environmental factors can interfere with the normal processes of DNA replication, transcription, and chromosome segregation, leading to genomic instability, cell aging, and/or apoptosis (Kusakabe et al., 2019). Several studies have demonstrated that the xenobiotic exposure is able to cause oxidative stress and inhibit algae growth (Nie et al., 2013). Oxidative stress originates from an imbalance between the generations of reactive oxygen species (ROS) and cellular antioxidant defenses. ROS can induce a number of covalent modifications to DNA, which encompass single-nucleobase lesions, strand breaks, inter- and intra-strand cross-links, along with protein-DNA cross-links (Melis et al., 2013). The accumulation of ROS in cells can lead to the peroxidation of membrane lipids, which produces malondialdehyde. ROS may also give rise to DNA damage, blocking the transmission of genetic information, leading to upregulation of the cell cycle (Zhu et al., 2018). The energy fixation and insufficient growth of algae (e.g., energy may be mainly used to buffer ROS) may severely inhibit DNA replication and repair (Guo et al., 2020). As the main defense system against these detrimental effects, organisms have evolved multiple DNA repair pathways, including nucleotide excision repair (NER), base excision repair (BER), and DNA mismatch repair (MMR) (Kusakabe et al., 2019). While multiple repair pathways are simultaneously involved in the oxidative DNA damage repair, BER is considered to be the dominating approach to repair the oxidative stress-induced DNA damage (Melis et al., 2013). NER is the main pathway responsible for the removal of bulky DNA lesions induced by UV irradiation, environmental mutagens, and certain chemotherapeutic agents (Schärer, 2013). In the present study, the downregulated DEGs including *pcna*, *pold1*, *pole*, *pole2*, *rfa1*, *rfa2*, and *rfc3/5* were involved in the repair and ligation in both pathways. The BER system is the most important repair mechanism of base DNA damage caused by oxidative agents. The BER process occurs in four core steps, covering excision of the base, incision, end processing, and repair synthesis (Krokan and Bjørås, 2013). The expression of the majority of DEGs enriched in this pathway, consisting of *fen1*, *pcna*, *pold1*, *pole*, and *pole2*, was decreased during the process of BER, leading to the poignant inhibition of BER. Previous studies have shown that the inhibition on BER was a response to salt stress in *Shewanella* algae (Fu et al., 2014). The relationship between BER damage and mutation in algal species remains to be poorly understood. MMR is a highly conserved DNA repair system that contributes to maintain genome stability through the correction of mismatched base pairs derived from replication errors (Fukui, 2010). In eukaryotes, the MMR system functions through a complex interaction among multiple proteins such as MutSα and MutSβ. In the high SMX treatment group, *msh2* in the MutSα and MutSβ recognizes that the initial error of DNA was downregulated (Table 1). *rfa1* is the component of the replication protein A complex (RPA), which is also a key component in multiple DNA repair pathways. RPA seems to be involved in all stages of MMR including the stimulation of mismatch-provoked excision and facilitation of DNA resynthesis (Li et al., 2019). The decrease of RPA suggested a weakened DNA repair capability. The inhibited DNA repair pathways suggested that genome-wide point mutation rates were increased because of the unrepaired DNA synthesis errors. Mutation is a central biological process whose rates and spectra are influenced by a variety of complex. In *Caenorhabditis elegans*, the importance of three excision repair pathways in maintaining the stability of the genome were as follows: MMR over NER over BER (Denver et al., 2006). Although DNA repair pathways may play crucial roles in maintaining genetic stability of algal species, the explicit relationship between these pathways and the mutation accumulation remain elusive.

### Genes Related to Photosynthesis

Chlorophyll widely presented in algal species is essential for light energy acquisition and transfer in photosynthesis. The synthesis of chlorophyll in the chloroplast initiates from the first fixed precursor 5-aminolevulinic acid (ALA), which is converted from L-Glutamyl-tRNA (Glu) catalyzed by Glu-tRNA reductase (*hemA*) and L-Glutamate-1-semialdehyde 2, 1 aminomutase (Hunsperger et al., 2015). Downregulation of both genes in the high SMX treatment group may lead to a lower ALA content. The enzyme-catalyzed reaction to synthesize ALA from Glu is the first stage of the plant chlorophyll biosynthetic pathway, determining the total flux of this pathway (Cornah et al., 2003). Subsequently, during the synthesis of porphyrinogen IX from ALA, the enzymes encoded by porphobilinogen synthase (*HemB*), hydroxymethylbilane synthase (*HemC*), uroporphyrinogen decarboxylase (*HemE*), and coproporphyrinogen III oxidase (*HemF*) were downregulated. Porphyrinogen IX enters the chlorophyll synthesis pathway through a magnesium chelation reaction, and finally, produces chlorophyll. Here, the genes encoding the key enzymes, including magnesium chelatase subunitH (*chlH*), magnesium-protoporphyrin O-methyltransferase (*chlM*), vitamin chlorophyllide an 8-vinyl-reductase (*dvr*), and protochlorophyllide reductase (*por*), were downregulated during the process. Intermediate protochlorophyllide (PChlide) also has a negative feedback mechanism for the synthesis of ALA. ALA synthesis has been attributed to a coordinated heme and PChlide signaling (Brzezowski et al., 2015). The reduction of PChlide is considered to be the rate-limiting step of the chlorophyll branch and as the rate of Chl protein synthesis depends strictly on Chl availability. Moreover, the shortage and excess of por enzyme activity will have a fatal effect on cell activity (Qin et al., 2020). Other pigments such as chlorophyll *b* and carotenoids absorb light energy and transfer to chlorophyll *a* (Rai et al., 2014). It is worth noting that the genes related to chlorophyll *b* and carotenoids synthesis were not differentially expressed, indicating that SMX targeted only the synthesis of chlorophyll *a* rather than that of chlorophyll *b* and carotenoids (Table 1). SMX-induced gene expression patterns were in accordance with the alteration at the physiological levels; the contents of ALA, protochlorophyllide, and chlorophyll *a* were decreased in *R. subcapitata* treated with SMX at the concentration of 0.5 mg L^–1^ (Liu et al., 2011a). Decreased chlorophyll may lead to decreased photosynthesis, resulting in the insufficient energy of algal cells, which may be an important reason for its growth inhibition (Guo et al., 2020). Moreover, the chlorophyll content in cells can also be used as one of the protective mechanisms to eliminate accumulated ROS (Xiong et al., 2016). Therefore, reduced chlorophyll content may affect the DNA replication and antioxidant capacity of algal cells.

Photosynthesis transforming sunlight into chemical energy occurs in and around the thylakoid membranes of green plants, algae, and photosynthetic bacteria (Chmeliov et al., 2016). This process is driven by photosystems I and II (PSI and PSII), which are large protein assemblies containing hundreds of pigments complexes. Pigments in pigment-protein complexes, known as light-harvesting complexes, are responsible for the absorption of sunlight (Mirkovic et al., 2017). The primary reactions of photosynthesis in plants are initiated at light harvesting. In the present study, chlorophyll *a/b* binding protein 2 (*lhca2*) in light-harvesting complex I and chlorophyll *a/b* binding proteins 2, 4, 5 (*lhcb2, 4, 5*) in light-harvesting complex II were downregulated by SMX, which may inhibit the absorption of light in photosynthesis. SMX blocks PSII electron transmission and further impedes the transmission of excitation energy from chlorophyll molecules (P680) to PSI (Liu et al., 2011b). The damaged electron transport chain may result in electron accumulation in the chloroplast, where oxygen molecules accept electrons to generate ROS that damages chloroplast in a continuous degradation cascade (Zhu et al., 2018).

### Genes Related to Translation

Ribosomes are the cellular machines that translate the genetic code in mRNA and catalyze protein synthesis in all organisms (Woolford and Baserga, 2013). In the high SMX treatment group, expression of a multitude of DEGs involved in the ribosome biogenesis pathway was completely repressed, suggesting an inhibited ribosomal synthesis. In particular, complete fibrillarin (*nop1*) and nucleolar protein 56 (*nop56*) are involved in the pre-rRNA 2′-O-ribose methylation-modification. *Nop1* is an important methyltransferase in eukaryotes and can covalently modify pre-rRNA (Lin et al., 2011). *Nop56* is part of the box C/D snoRNP complex that directs 2′-O-methylation of pre-rRNA during its maturation (Lykke-Andersen et al., 2018). Inhibition of these two genes may thus interfere with the maturation of pre-rRNA and even the stability of RNA secondary structure, disrupting ribosome formation and/or reducing ribosome translation efficiency. *Rcl1* delivered to pre-ribosomes by ribosome biogenesis protein *BMS1* is required for co-transcriptional cleavage during 18S rRNA biogenesis (Horn et al., 2011; Tanaka et al., 2011). Therefore, the downregulation of *Rcl1* may affect the formation of 18S rRNA. GTP-binding nuclear protein Ran (*ran*) binds to a conserved superfamily of nuclear transport receptors and facilitates the passage of the nuclear pores of proteins, RNA, and ribonucleoproteins in all eukaryotes (Merkle, 2011). Downregulation of *ran* may suppress the nuclear export and hinder the formation of the 40S small subunit. Ribosome biogenesis is closely tied to the growth and proliferation of cells (Donati et al., 2012). While complete function loss mutations of assembly in most factors and ribosomal proteins cause yeast and embryonic mortality (Woolford and Baserga, 2013), the relationship between the SMX-inhibited ribosome assembly and algae cell survival remained poorly understood.

Aminoacyl-tRNA synthesis that faithfully translates the genetic information from mRNA to protein is critical for normal cellular function (Ling et al., 2009). During the transfer of amino acids to tRNA in aminoacyl-tRNA synthesis, several aminoacyl-tRNA synthetase encoding genes including glutamyl-tRNA synthetase (*gltx*), leucyl-tRNA synthetase (*leus*), lysyl-tRNA synthetase (*lysk*), and tyrosyl-tRNA synthetase (*tyrs*) were downregulated in the high SMX treatment group. Oxidative stress disrupts the function of aminoacyl-tRNA synthetases, leading to the misfolding of proteins and damage to cells (Ling and Söll, 2010). Severe oxidative stress induces protein mistranslation through impairment of an aminoacyl-tRNA synthetase editing site (Ling and Söll, 2010). Naturally occurring aminoacyl-tRNA synthetases editing-domain mutations that cause mistranslation in Mycoplasma parasites (Li et al., 2011). Since the aminoacyl-tRNA synthetase determines the genetic code by precisely matching homologous tRNA with its corresponding amino acid during the translation, the inhibition of aminoacyl-tRNA synthetase may lead to an abnormal protein structure (Lin et al., 2011).

## Conclusion

To sum up, SMX at an environmentally relevant exposure did not remarkably inhibit the growth or alter the transcriptional profile in *R. subcapitata*. Contrarily, exposure to a high concentration of SMX inhibited the growth by approximately 63%. At the transcriptomic level, DNA replication and repair processes, chlorophyll, photosynthesis, and ribosome biogenesis were downregulated. These suggested that SMX may cause DNA damage and inhibit the translation process in *R. subcapitata*. However, DEGs were not enriched in folate biosynthesis, suggesting that the mode of action of antibiotics in bacteria may not directly be extrapolated to green algae. Overall, the present study gained novel insights into the effects of SMX on algae and demonstrated that transcriptomics is an effective approach for predicting the adverse outcomes at the physiological and individual levels.

## Data Availability Statement

The datasets generated for this study can be found in the National Omics Data Encyclopedia (NODE) under accession number OEP001463 (https://www.biosino.org/node/index).

## Author Contributions

JG: conceptualization, methodology, writing of the original draft, and funding acquisition. YZ: methodology, validation, formal analysis, investigation, data curation, and writing of the original draft. JM: methodology, writing—review and editing, and formal analysis. HS: writing—review and editing and supervision. QL: writing—review and editing, supervision, and project administration. All authors reviewed the final manuscript.

## Conflict of Interest

The authors declare that the research was conducted in the absence of any commercial or financial relationships that could be construed as a potential conflict of interest.

## Footnotes

1 https://www.biosino.org/node/index
