# Supplementary material for: Sulfamethoxazole-Altered Transcriptomein Green Alga Raphidocelis subcapitata Suggests Inhibition of Translation and DNA Damage Repair
Source: Front Microbiol. 2021 Jul 19;12:541451. doi: 10.3389/fmicb.2021.541451 (PMC8326373; doi:10.3389/fmicb.2021.541451)
Supplement: Supplementary file 1 [file Table_1.DOCX]

Supplementary Material

## SPE methods and processes

The actual SMX concentration of each treatment was determined by solid phase extraction (SPE) followed by a 1290 series liquid chromatograph coupled with an Agilent series 6470 triple quadrupole mass spectrometer device (LC-MS/MS, Agilent, USA). The collected samples were diluted to 50 mL with culture medium, and each sample was spiked with 30 ng of Sulfamethoxazole-13C6, as the internal surrogate standard. Samples were extracted by pressurized liquid extraction using solid phase extraction unit (Shanghai Nai Precision Instrument Co., Ltd.) Placed the Oasis® HLB solid phase extraction cartridge (3cc, 60 mg, Waters, USA) on the extraction unit and then activated with 3 mL of methanol and 3 mL of ultrapure water. The sample solution was passed through the cartridge at flow rate of 5–10 mL/min, rinsed the beakers by 10 mL ultra-pure water after samples were all passed through the cartridges, and then evacuated for one hour. After that, the analyte was eluted into the test tube four times with methanol, 3 mL each time. Under a 37°C water bath, dry under a nitrogen stream. The samples of different concentration were reconstituted with Different amounts of methanol (low concentration: 0.5 mL. medium concentration and blank group: 1 mL. High concentration: 3 mL). Then Added different volume of Atrazine-d5 internal standards (IS) to these four groups, and made the concentration of Atrazine-d5 in each sample is 20 ng mL^−1^. Mixed well and filtered with 0.22 μm micron nylon membrane. The sample solution was then transferred to an autosampler vial (with insert) for LC/MS/MS analysis.

## Quantitative real-time PCR analysis

We conducted the relative quantitation of gene expression using Real-time PCR (TIB8600, Triplex International Biosciences, China). Initially, total RNA was reverse transcribed into cDNA using the PrimeScript TM 1st stand cDNA Synthesis Kit. Gene-specific qRT-PCR primers for mRNA quantification in *R. subcapitata* were as follows: *pcna-fwd*: 5’-AACATCGACGTGAGCAGCAG-3’, *pcna*-rev: 5’-TTGGCCTTGAGCGTCACAAT-3’; *fen1-fwd: 5’-GAGTGCATCAAGGAGACAAA-3’, fen1-rev: 5’-CACGATCAGGAACTGGTAGA-3’; ChlM*-fwd: *5’-TCAAGACCTACTTCAAC*ACCAG-3’, *ChlM*-rev*:* 5’-CCTCCTCGTCAAACCACTT*-3’. mcm2-fwd*: 5’-CTGACGGACGAGGACAAG-3’, *mcm2*-rev: 5’- CGAGGGAGGTCTTGATGTT-3’.

Four primers were synthesized by Shanghai Personal Biotechnology Co., Ltd. (China). Each 20 μL reaction mix was consisted of 10 μL 2× SYBR real-time PCR premixture, 0.4 μL forward primer (10 μM), 0.4 μL reverse primer (10 μM), 1 μL cDNA, and 8.2 μL RNase free dH2O. Reactions were performed in triplicate in the following conditions: thermally denatured at 95°C for 5 min followed by 40 cycles consisting of 95°C for 15 s and 60°C for 30 s. With the temperatures ranging from 60°C to 95°C, melting curve analysis was conducted for all samples at the end of each run. The mRNA levels for each target gene were calculated with the 2-ΔΔCt method and normalized to a housekeeping gene *ubc*. For each gene, expression of SMX-treated samples relative to that of controls was estimated.


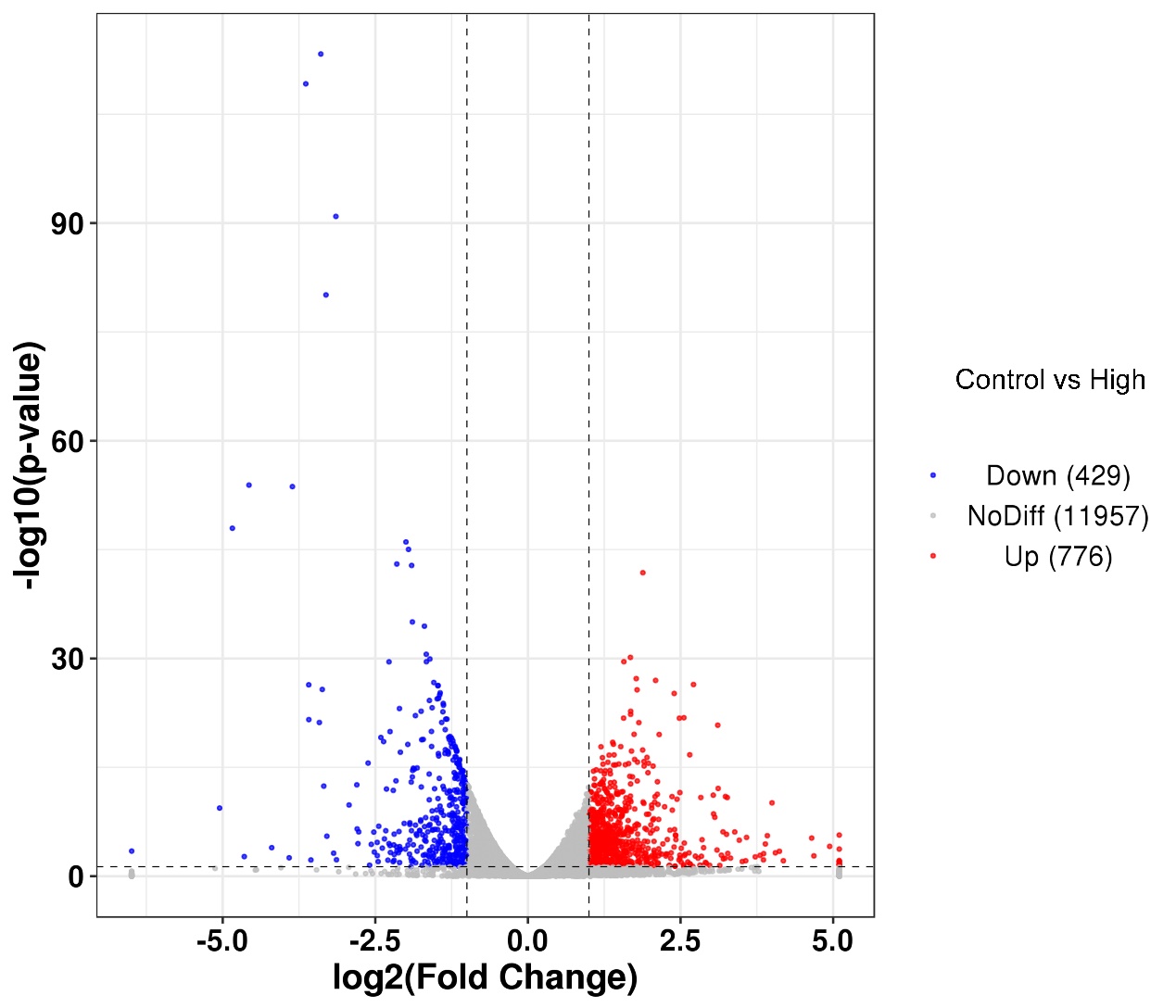


**Supplementary Figure 1**. Volcano plot of gene expression profile in the high treatment group.

Control vs Low


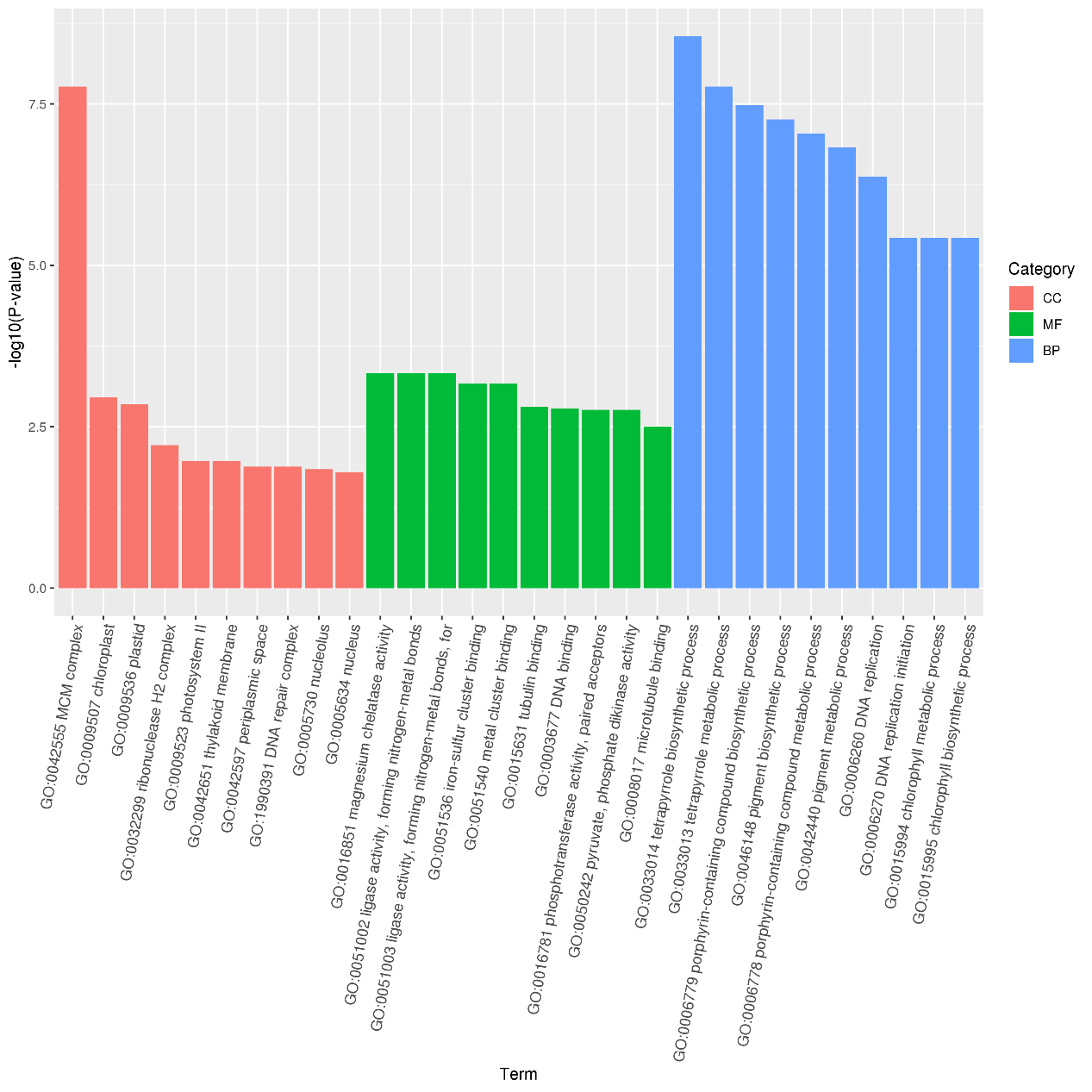


Control vs High


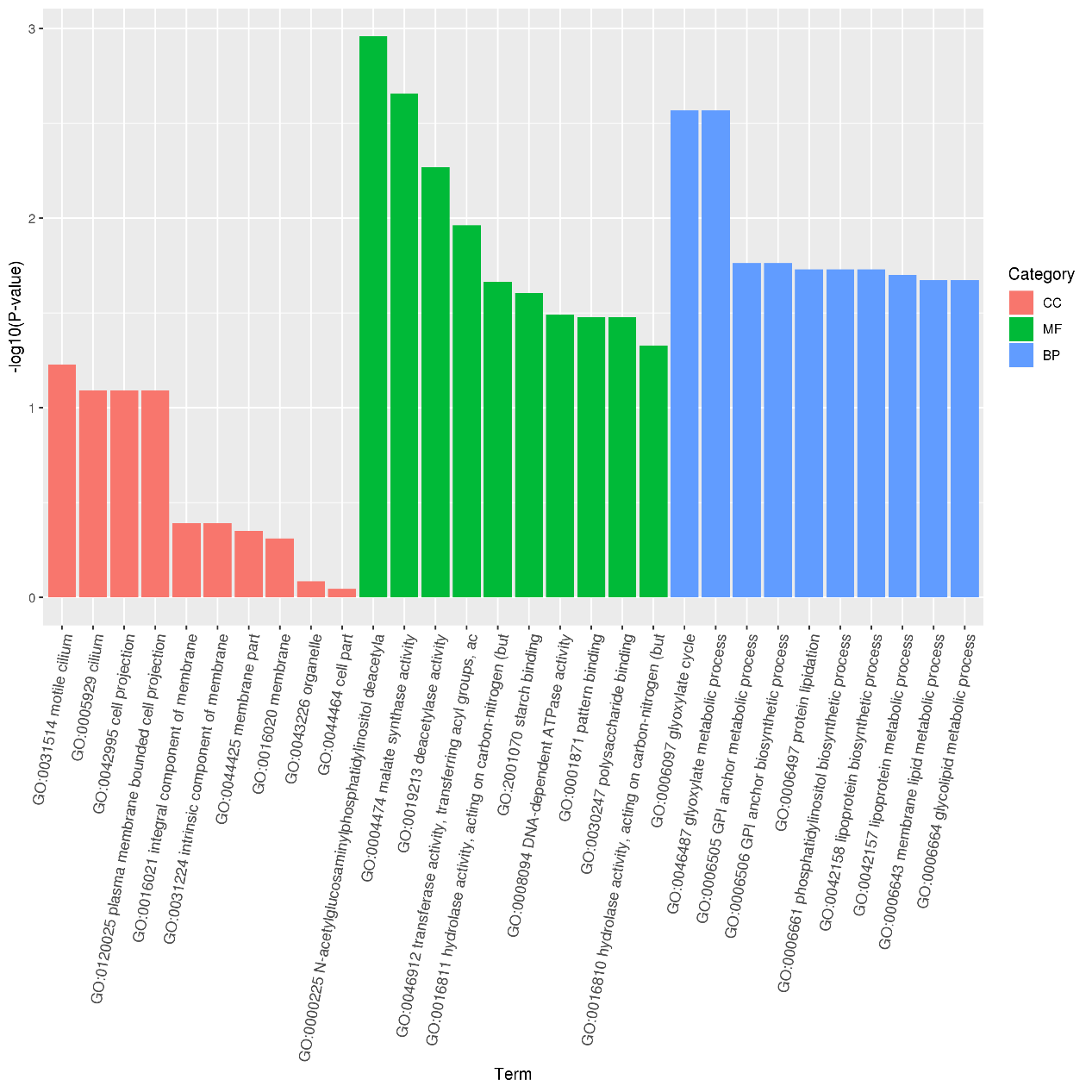


**Supplementary Figure 2.** GO enrichment analysis of differentially expressed genes (DEGs) in two SMX treated groups.

**Supplementary Table 1.** HPLC program on the condition of positive electrospray ionization.

| **Column** | **Agilent ZORBAX Eclipse Plus C18 HPLC column (3×100 mm, 1.8 μm),** | | |
| --- | --- | --- | --- |
| Mobile phase | A: ultrapure water with 0.1% formic acid (v/v) | | |
|  | B: Acetonitrile | | |
| Column Temperature | 24°C |  |  |
| Injection volume | 10 μL |  |  |
| Flow rate | 0.3 mL/min |  |  |
| Gradient | Time (min) | A (%) | B1(%) |
|  | 0.00 | 92.5 | 7.5 |
|  | 1.00 | 92.5 | 7.5 |
|  | 3.00 | 88.0 | 12.0 |
|  | 4.50 | 80.0 | 20.0 |
|  | 6.00 | 40.0 | 60.0 |
|  | 9.00 | 10.0 | 90.0 |
|  | 10.00 | 10.0 | 7.5 |
|  | 11.00 | 92.5 | 7.5 |

**Supplementary Table 2.** Optimized retention time, ion transitions, ion transitions, collision cell exit potential for MS/MS determination of target antibiotic.

| **Compound** | **Retention time (min)** | **Ion transitions (m/z)** | **Collision energy (eV)** | **Collision cell exit potential (V)** |
| --- | --- | --- | --- | --- |
| Sulfamethoxazole | 7.06 | 254 > 155.9  254 > 108 | 80 | 15  25 |
| Sulfamethoxazole-13C6 | 7.073 | 283.1 > 124.2  283.1 > 186.1 | 124 | 30  20 |
| Atrazine-d5 | 8.579 | 221.0 > 101.0  221.0 > 137.0  221.0 > 179.0 | 113 | 30  25  20 |

**Supplementary Table 3.** Actual concentration of SMX in algal medium.

| **Algal** | **SMX concentration (mg L^−1^)** | **Actual concentration of SMX (mg L^−1^)** |
| --- | --- | --- |
| *R. subcapitata* | 0  0.005  0.3 | 0.000  0.0047  0.26 |
